# Endoscopists attitudes on the publication of "quality" data for endoscopic procedures: a cross-sectional survey

**DOI:** 10.1186/1471-230X-7-30

**Published:** 2007-07-24

**Authors:** Sarah A Hearnshaw, Helena M Maddock, David Nylander, Martin I Prince

**Affiliations:** 1 NHS Blood and Transplant, John Radcliffe Hospital, Oxford, UK; 2 Gastroenterology, Sunderland Royal Hospital, Sunderland UK; 3 Gastroenterology, Manchester Royal Infirmary, Manchester, UK

## Abstract

**Background:**

Whilst the public now have access to mortality & morbidity data for cardiothoracic surgeons, such "quality" data for endoscopy are not generally available. We studied endoscopists' attitudes to and the practicality of this data being published.

**Methods:**

We sent a questionnaire to all consultant gastrointestinal (GI) surgeons, physicians and medical GI specialist registrars in the Northern region who currently perform GI endoscopic procedures (n = 132). We recorded endoscopist demographics, experience and current data collection practice. We also assessed the acceptability and utility of nine items describing endoscopic "quality" (e.g. mortality, complication & completion rates).

**Results:**

103 (78%) doctors responded of whom 79 were consultants (77%). 61 (59%) respondents were physicians. 77 (75%) collect any "quality" data. The most frequently collected item was colonoscopic completion rate. Data were most commonly collected for appraisal, audit or clinical governance. The majority of doctors (54%) kept these data only available to themselves, and just one allowed the public to access this. The most acceptable data item was annual number of endoscopies and the least was crude upper GI bleeding mortality. Surgeons rated information less acceptable and less useful than physicians. Acceptability and utility scores were not related to gender, length of experience or current activity levels. Only two respondents thought all items totally unacceptable and useless.

**Conclusion:**

The majority of endoscopists currently collect "quality" data for their practice although these are not widely available. The endoscopists in this study consider the publication of their outcome data to be "fairly unacceptable/not very useful" to "neutral" (score 2–3). If these data were made available to patients, consideration must be given to both its value and its acceptability.

## Background

Until recently, members of the public had virtually no access to data about the performance of individual doctors in the United Kingdom. Increased choice within healthcare systems and several high profile cases have lead to calls for such data to be more freely available [1]. The inquiry into paediatric cardiac surgery at Bristol Royal Infirmary recommended "that patients must be able to see information about the relative performance of individual consultants" [2]. In response to this Bridgewater reported adult mortality rates for named cardiac surgeons from Northwest England [3] and more recently the healthcare commission published further national cardiothoracic data [4]. Even in the USA, the data, available for other surgical specialties, is limited in scope and of poor quality [5].

For endoscopy, Cotton has promoted the use of continuously updated "report cards" containing quality parameters provided either routinely or on request [6]. An expert group suggested these should include individual endoscopist's experience and current case complexity, outcomes, and adverse events [7]. However presently, with the exception of colon cancer screening, there are no statutory requirements in the UK for consultant endoscopists to collect endoscopy outcome data.

In light of the changing patient expectations and increasing public awareness of rights to information, we decided to find out the opinions of endoscopists on the usefulness and practicality of making endoscopist 'quality outcome data' available to the public in the UK.

## Methods

### Study design

We designed a questionnaire to perform this cross sectional survey of endoscopists. The questionnaire was not validated or piloted. The full questionnaire is available as Additional file 1.

### Setting and participants

We posted the questionnaire (with a single reminder for non-responders) to all consultant gastroenterologists, surgeons and medical gastroenterology specialist registrars in the Northern Region of England who currently perform gastrointestinal endoscopy. Subjects were identified from a telephone survey of all endoscopy units.

### Data collection

We recorded basic demographics, level of experience, grade, and current activity levels including the range of procedures undertaken. Respondents answered questions about their current data collection practice including the type and purpose of any data collected. We also asked whether any data was made available to the public.

Respondents were asked to assess the utility and acceptability of nine items (see table 3 for details) describing endoscopic outcomes including mortality, success rates and complication rates. Attitudes were assessed using a five point Likert scale (higher scores corresponding to the data being perceived as more acceptable or more useful).

**Table 3:** Acceptability and utility scores for nine items of endoscopic outcome data

**Data item**	**Mean acceptability score (SD)**		**Mean utility score (SD)**	
1. 30-day mortality after all endoscopic procedures	2.54	(1.46)	2.05	(1.44)
2. Crude in patient mortality after OGD for upper GI haemorrhage	2.05	(1.31)	1.83	(1.25)
3. Rockall adjusted in patient mortality after OGD for upper GI haemorrhage	2.70	(1.22)	2.44	(1.22)
4. Crude colonoscopy caecal intubation rate	2.55	(1.39)	2.17	(1.32)
5. Colonoscopy caecal intubation rate adjusted for "unavoidable" failure – e.g. obstructive tumours etc	3.10	(1.23)	2.81	(1.24)
6. ERCP (intended duct) cannulation rate	2.92	(1.17)	2.72	(1.20)
7. ERCP completion rate	2.93	(1.14)	2.71	(1.20)
8. ERCP complication rate	2.92	(1.19)	2.72	(1.21)
9. Numbers of endoscopic procedures performed annually	3.10	(1.17)	2.67	(1.18)

**Acceptability Likert scale: **1 – Very unacceptable, 2 – Fairly unacceptable, 3 – Neutral, 4 – Fairly acceptable, 5 – Very acceptable

**Utility Likert scale **1 – Not useful at all, 2 – Not very useful, 3 – Neutral, 4 – Fairly useful, 5 – Very useful

### Methods of analysis

Analysis was performed on SPSS using non-parametric methods (Mann-Whitney U test).

## Results

We sent the questionnaire to 132 doctors of whom 103 (78%) responded. The response rate was higher from medical gastroenterology consultants (39 out of 44 (89%)) than surgical (40 out of 61 (66%)). Table 1 shows the characteristics of respondents. The vast majority of respondents performed both gastroscopy and colonoscopy, with most doing more than 100 procedures annually. 16 of the respondents who performed endoscopy for acute gastrointestinal bleeding only did so under supervision. All 16 were specialist registrars in medical gastroenterology.

**Table 1:** Characteristics of respondents to survey

**Characteristic**		**Number**	**(%)**
Current post	Total	103	
	Consultant surgeon	40	(39%)
	Consultant physician	39	(38%)
	Registrar in gastroenterology	22	(21%)
	Other	2	(2%)
Time since qualification (years)	Median (Inter Quartile Range) (IQR)	18	(13–24)
Time in current post* (years)	Median (IQR)	6	(4.8–16)
Procedures currently performed (number of respondents performing)			
	Gastroscopy	99	(96%)
	Colonoscopy	94	(92%)
	Flexible sigmoidoscopy	92	(89%)
	ERCP^†^	22	(21%)
	Treatment of GI haemorrhage	82	(80%)
Annual no. of procedures performed median (IQR)			
	Gastroscopy	200	(100–341)
	Colonoscopy	150	(70–250)
	Flexible sigmoidoscopy	50	(30–100)
	ERCP	80	(60–116)

*Consultants only included

^†^Two respondents did not answer question

77 (75%) respondents formally collected any data on their endoscopy performance. As shown in table 2, this rate was higher in physicians than surgeons (p < 0.001), and highest amongst specialist registrars. Doctors performing ERCP were also more likely to collect data. There were no associations between current endoscopy activity levels, gender, or consultant level of experience (indicated either by length of time in post or time since qualification) and the proportion of doctors collecting data (not shown).

**Table 2:** Proportions of different professional groups that collect data

Professional Group (total number)	No collecting data	(%)	p
Consultant surgeons (40)	16	(40)	
Consultant physicians (39)	34	(87)	P < 0.001
Registrars in gastroenterology (22)	21	(95)	
Currently performing ERCP (22)	21	(95)	P = 0.008
Not currently performing ERCP (77)	53	(69)	
Currently treating upper GI haemorrhage (82)	64	(78)	P = 0.130
Not currently treating upper GI haemorrhage (21)	13	(61)	

The most frequently collected data were colonoscopy completion rates, collected by 57 endoscopists. ERCP cannulation rates (recorded by 7 respondents) and complication rates (2 respondents), sedation complications (6 respondents), and problems with bowel preparation (2 respondents) were the other most frequently collected data.

Data were collected for organised audit by 26 doctors (34% of those collecting data) for clinical governance by 30 doctors (39%) and for personal appraisal by 62 doctors (81%). 34 respondents said data were routinely accessible by other staff. Only one respondent reported that patients could currently access their data.

Table 3 shows respondents attitudes to publication of differing items of "quality" data. Acceptability scores were higher than utility scores for all information items (p < 0.001). There were statistically significant differences between the data items both for acceptability (non-parametric ANOVA T = 49, p < 0.001) and utility scores (T = 59, p < 0.001). The data items rated as most acceptable were the annual number of endoscopies performed and "adjusted" caecal intubation rates (mean acceptability scores of 3.10). The most useful data item was the "adjusted" colonoscopy caecal intubation rate (mean utility score 2.81). The data item considered both least acceptable and least useful was the crude in-patient mortality rate after OGD for upper GI haemorrhage, with a mean acceptability score of 2.05 and utility score of 1.83. Several respondents made hand written comments on the questionnaire that mortality rates reflected multiple facets of the organisation of endoscopy services and not just endoscopists' performance.

Overall consultant surgeons rated information as being less acceptable and less useful than consultant physicians with a mean acceptability score of 2.1 vs 3.1 (p < 0.01), and a mean utility score of 2.0 vs 2.6 (p < 0.01). Specialist registrars thought data more useful than did consultants with a mean utility score of 3.0 vs 2.3 (p < 0.01). Acceptability and utility scores were not related to endoscopists gender, length of consultant experience or current activity levels (measured by either total number of procedures or numbers of individual procedures) (all p > 0.05). Only two respondents (both consultant surgeons) rated all items as being totally unacceptable and useless.

## Discussion

Data comparing the relative performance of individual doctors and hospitals is likely to be more easily accessible to colleagues and patients in the future. We aimed to investigate what information endoscopists currently collect and attitudes to the publication of such data. As a cross sectional regional survey it provides a snapshot of practice from both district and tertiary hospitals. The high response rate may reflect the relevance and importance of this issue to practising endoscopists. The high response rate minimised the potential for responder bias caused by those least in favour of data publication being less likely to respond.

Encouragingly, the majority of consultant endoscopists already collect some data on their practice. These data are frequently collected for governance or appraisal. However, very few respondents made this information available to colleagues and only one stated the public had access to it. The type of data collected was variable and although colonoscopy completion rates were the most frequently collected, these could be inconsistently assessed (for example whether or not correction is made for procedures that were not completed due to poor preparation). By comparison, virtually all registrars collected such data probably because it is routinely reviewed in annual training assessments.

To our knowledge this study is the first to address endoscopists' attitudes to such data being published. The acceptability and utility questionnaire used here is not a previously validated tool. The data items were selected to reflect areas where information might be easily collectable and possible to present in a numerical format. However, the information that is easiest to collect may not be the most important or relevant, as evidenced by comments regards mortality rate. Validation work is necessary to see which factors are truly the most reflective of the "quality" of the service. Cotton et al [7] published a large number of potential "metrics of excellence" after this study was undertaken, including activity levels, success rates, complication rates, and for colonoscopy – polypectomy rates and time spent in extubation. In the United Kingdom, more detailed process data for example departmental organisation, patient satisfaction scores, waiting time etc. have been audited using the endoscopy global rating scale [8]. Further work is necessary to assess the value of publication of these data.

Very few respondents thought that publication of these data was totally unacceptable and useless although there were differences between scores for different data items. The items considered least acceptable and least useful data were mortality data. Endoscopists may feel these data least reflect their skill as an endoscopists because they reflect more general issues regarding organisation and provision of services [9]. It is important that a framework for interpreting these data is thus provided to help non-specialists interpret the significance of any variation [10].

We did not attempt to identify what data patients actually want or how useful this would be. Whilst public surveys suggest that health provider data is rated as being important, it is not clear how useful this data is [11]. Patients are still far more likely to rely on recommendations from the referring doctor than to use data made available on the internet [12]. Patients undergoing surgery rate factors such as accessibility of the hospital, delays to treatment and nursing ratios as far more important than outcome data [12,13].

## Conclusion

In summary this study shows most endoscopists currently collect "quality" data for their practice although these are not currently available to referrers or the public. The endoscopists in this study consider the publication of their outcome data to be "fairly unacceptable/not very useful" to "neutral" (score 2–3). It is likely these data will soon be demanded by both patients and governance organisations. Clinicians need now to address how best to diseminate the most useful outcome data in a clear and meaningful way, and in a way that is acceptable to them. For the data to be interpretable by the public, they would need to be collected in a uniform way and further work is needed to assess what information patients and referring doctors want and would find useful. It is also important to assess the attitudes of nurse and GP endoscopists.

## Competing interests

The author(s) declare that they have no competing interests.

## Authors' contributions

SAH – contributed to the design of the study and the questionnaire, co-ordinated data collection and co-wrote the manuscript

HM – contributed to the design of the study, helped in data collection and with the preparation of the manuscript

DN – contributed to the design of the study and the questionnaire, and contributed to the manuscript preparation

MP – had the idea for the study, designed the questionnaire, entered the data, carried out the statistical analysis and co-wrote the manuscript

All authors read and approved the final manuscript

## Pre-publication history

The pre-publication history for this paper can be accessed here:



## Supplementary Material

Additional file 1 Public data questionnaire 4. The survey questions used in the study are provided Click here for file
