# Supplementary material for: Endoscopists attitudes on the publication of "quality" data for endoscopic procedures: a cross-sectional survey
Source: BMC Gastroenterol. 2007 Jul 24;7:30. doi: 10.1186/1471-230X-7-30 (PMC1950092; doi:10.1186/1471-230X-7-30)
Supplement: Additional file 1 — Public data questionnaire 4. The survey questions used in the study are provided [file 1471-230X-7-30-S1.doc]

# Part 1: Demographics

**What is your current grade?**

- - - 1. Consultant
      2. Specialist registrar
      3. Other

**What year did you qualify?** 19 __ __

**How many years have you been in you current grade?** __ __

**What is your main speciality?**

1. A medical gastroenterologist
2. A surgical Gastroenterologist
3. Other specialty performing endoscopy

**What is your gender?**

- - - 1. Male
      2. Female

**How many endoscopic procedures did you perform**

**in the last 12 months?**

Gastroscopy __ __ __

Colonoscopy __ __ __

Flexible sigmoidoscopy __ __ __

ERCP __ __ __

## Do you currently perform therapeutic endoscopies for

**upper GI haemorrhage**

- - - 1. Yes
      2. Yes with supervision
      3. No

Part 2: Current data available

**Do you currently formally collect data on any marker of endoscopic**

**“quality” (e.g. colonoscopy completion rates)?**

- - - 1. Yes
      2. No

**What data do you currently collect?**

……………………………………………………………………….

……………………………………………………………………….

……………………………………………………………………….

## How is this data collated?

1. On an individual basis
2. Averaged across team members

**Why is this data collected?**

- - - 1. Organised audit
      2. Clinical governance
      3. Personally collected for appraisal etc
      4. Other (please specify)

…………………………………………………………………..

…………………………………………………………………..

**Who may access this data?**

- - - 1. Only you & those collecting data
      2. Other staff (e.g. colleagues)
      3. Patients

# Part 3: Views on future data collection

For each of the following items of data, answer on the scale below how acceptable you would find it for this data to be publicly available & whether you feel such data would be useful to those who may access it (e.g. patients, GP’s, colleagues).

**Acceptability**  **Utility**

1. Very unacceptable 1. Not useful at all
2. Fairly unacceptable 2. Not very useful
3. Neutral 3. Neutral
4. Fairly acceptable 4. Fairly useful
5. Very acceptable 5. Very useful

**Acceptability Utility**

30-day mortality after all endoscopic procedures 1 2 3 4 5 1 2 3 4 5

Crude in-patient mortality after OGD for upper GI haemorrhage 1 2 3 4 5 1 2 3 4 5

Rockall adjusted in patient mortality after OGD for upper GI haemorrhage 1 2 3 4 5 1 2 3 4 5

Crude colonoscopy caecal intubation rate 1 2 3 4 5 1 2 3 4 5

Colonoscopy caecal intubation rate adjusted for “unavoidable”

failure – e.g. obstructive tumours etc 1 2 3 4 5 1 2 3 4 5

ERCP (intended duct) cannulation rate 1 2 3 4 5 1 2 3 4 5

ERCP completion rate 1 2 3 4 5 1 2 3 4 5

ERCP complication rate 1 2 3 4 5 1 2 3 4 5

Numbers of endoscopic procedures performed annually 1 2 3 4 5 1 2 3 4 5
